# Rational Design of Deep Eutectic Solvent-Mediated MOF-Based Membranes for the Recovery of Pb(II) and Cr(III) Ions Toward a Circular Economy

**DOI:** 10.3390/membranes16060205

**Published:** 2026-06-10

**Authors:** Saif-ur-Rehman, Urooj Ahmad, Muddasar Jamal, Arafat Husain, Bart Van der Bruggen, Ali H. Al-Marzouqi

**Affiliations:** 1 Department of Chemical Engineering, ProcESS-Process Engineering for Sustainable Systems, KU Leuven, Celestijnenlaan 200F, B-3001 Leuven, Belgium; saifurrehman.saifurrehman@kuleuven.be (S.-u.-R.); urooj.ahmad@kuleuven.be (U.A.); muddasar.jamal@kuleuven.be (M.J.); arafat.husain@kuleuven.be (A.H.); 2 Department of Chemical and Biochemical Engineering, Korea University, 145 Anam-ro, Sungbuk-gu, Seoul 02841, Republic of Korea; 3 Chemical and Petroleum Engineering Department, United Arab Emirates University, Al Ain 15551, United Arab Emirates; hassana@uaeu.ac.ae

**Keywords:** deep eutectic solvents, metal–organic frameworks, mixed matrix membrane, polysulfone, Pb and Cr ion removal

## Abstract

The sustainable recovery of high-value metals from wastewater has garnered significant attention in light of the circular economy and environmental preservation. Because of its appealing characteristics, membrane separation technology is essential for the sustainable and effective recovery of valuable metals from wastewater, in contrast to conventional methods, which are chemical- or energy-intensive. In this study, a rational design approach was utilized to synthesize a metal–organic framework (MOF) using a deep eutectic solvent (DES) as a mediating medium to control the reaction of framework formation and particle properties. While DESs have been widely used for the physical modification of materials, their role as a chemically modifying medium during MOF synthesis for structural tailoring remains less explored. This synthesized MOF (DM-Zn-PDC@MOF) was further introduced as filler in polysulfone (PSf)-based mixed matrix membranes (MMMs). The performance of DM-Zn-PDC@MOF within the polymer matrix was examined. Several characterization techniques were used to thoroughly analyze the morphological, chemical, and physical characteristics of the MMMs and DM-Zn-PDC@MOF. The addition of the filler material significantly enhanced the membrane characteristics, including pure water flux, hydrophilicity, porosity, surface roughness, pore size, and heavy metal resource recovery in comparison with the pristine membrane. Stable incorporation of the filler within the membrane matrix was indicated by much less filler leaching (<5%) at all concentrations. With DM-Zn-PDC@MOF loading, the pure water flux increasedmore than nine times from 102.8 L/m^2^h (M-0) to 971.5 L/m^2^h (M-4). The functionalized membranes showed better flux retention in high-value heavy metal resource recovery using simulated wastewater: 871.8 L/m^2^h when filtering a Pb(II) ion solution (compared to M-0 with flux 120.6 L/m^2^h) and 526.8 L/m^2^h when filtering a Cr(III) ion solution (compared to M-0 with flux 97.1 L/m^2^h). These values represented approximately 7-fold and 5-fold improvements, respectively. Overall, Pb^+2^ > Cr^+3^, but the rejection of Cr(III) ions was also improved, when compared with M-0. The high flux of the membrane makes it easier to process large volumes and concentrate metals in the retentate, turning diluted contaminated streams into a concentrated feedstock for subsequent recovery procedures.

## 1. Introduction

Wastewater containing valuable metals is continuously released into the environment by various industries [1]. Because high concentrations of high-value metals are toxic, so their presence in wastewater can lead to health and environmental issues [2]. However, due to their remarkable physicochemical properties, high-value metals have a wide range of potential industrial applications [3]. Therefore, the limited supply of high-value metals, their high cost, and the difficulty of supplementation hinder the ability of these industries to expand globally [4]. Given these challenges, recovering high-value metals from industrial wastewater streams offers a practical alternative to sourcing them sustainably from natural resources [5]. By lowering the reliance on primary metal resources and minimizing environmental impacts, this strategy supports the circular economy. By extending the useful life of materials, the circular economy is a sustainable economic model that seeks to reduce waste and increase resource efficiency. The circular economy encourages the concepts of reduction, reuse, recycling, and recovery in contrast to the conventional linear strategy of “take–make–dispose.” This idea focuses on turning waste streams into useful resources in the context of wastewater treatment [6]. High-value metals can be extracted and recovered from wastewater using a variety of methods, including adsorption [7], ion exchange resins [8], electrocoagulation [9], leaching [10], solvent extraction [11], precipitation [12], and electrochemical methods [13] and by using ionic liquids [14]. However, these techniques have drawbacks, such as producing excess waste and using additional chemicals [1]. The intricate wastewater mixture presents another challenge, making it more difficult to extract specific valuable species.

In order to recover metals from complex wastewater effluents and turn waste into treasure, highly selective and chemical-free techniques are required. One of the best methods for removing valuable metals from wastewater streams is membrane technology because of its many advantages, including a high separation efficiency, low carbon footprint, ease of use, minimal phase change, low energy consumption, minimal space requirements, reduced chemical usage, and minimal solid waste generation [15]. Wastewater must first be pretreated before certain high-value metals can be recovered using selectively permeable membranes [16]. To capture specific high-value metals of interest, polymer membranes with different pore diameters and surface characteristics are used [17]. Compared to conventional techniques, the membrane-based approach has several benefits, including the ability to recover metals from low-concentration streams, environmental friendliness, and the selective recovery of particular valuable resources [18]. Membrane processes have some intrinsic drawbacks, despite their high separation efficiency. The creation of a concentrated retentate stream that contains accumulated contaminants necessitates additional treatment, which is a significant disadvantage that may raise operating expenses and environmental risks. Organic matter, scaling, or particulate deposition can cause membrane fouling, which lowers permeability and calls for regular cleaning or replacement. Furthermore, membrane processes can be energy-intensive [19], especially when operating under high pressure, and their durability under challenging industrial conditions may be limited by long-term chemical and mechanical stability.

Even though polymer-based materials have demonstrated appropriate qualities to be used as metal ion adsorbents, like easily regenerable surfaces, environmental compatibility, and customizable surface functionalities, they typically have a low adsorption capacity and selectivity [20]. One useful tactic for enhancing the performance of polymer matrices is the addition of porous nanomaterials [21]. In this context, metal–organic frameworks (MOFs) are porous crystalline materials [22] that have demonstrated an exceptional performance in recovering precious metals and selectively capturing specific inorganic contaminants [23]. Because of their many important benefits, including high water and structural stability [24] and easily functionalized microporosity that produces a broad range of pore sizes and shapes [25], along with the ability to be flexible and adaptable, MOFs are regarded as a promising and effective technology for removing heavy metals from water. These key advantages may be crucial in capturing and accommodating the targeted guest compound. However, there are certain drawbacks to using MOFs as nanocrystalline powders, such as handling difficulties, poor suspension stability, and the possible dispersion of the nanoparticles in water. For these reasons, their structures are very suitable for practical uses. In this regard, adding MOFs as fillers to polymeric matrices has produced a potent solution that gives MOF-embedded MMMs (MOFs-MMMs) an excellent performance and efficiency for wastewater treatment [26]. Chen et al. [27] created a β-cyclodextrin/zeolitic imidazolate framework-8 (β-CD@ZIF-8) nanoparticle-embedded polyvinylidene fluoride (PVDF) composite membrane that achieved saturation adsorption capacities of 708.13 mg/g for Pb^2+^ and 651.38 mg/g for Cu^2+^. Wang et al. [28] fabricated UiO-66-incorporated polysulfone (PSf) ultrafiltration membranes with improved hydrophilicity and antifouling performance (FRR ≈ 88%), as well as the efficient simultaneous removal of heavy metal ions (Sr^2+^, Pb^2+^, Cd^2+^, and Cr^2+^) and humic acid (≈93% rejection). Gu et al. [29] synthesized an NH_2_-MIL-53 immobilized wood–carbon (NH_2_-MIL-53/WC) hybrid membrane that demonstrated remarkable Pb^2+^ sequestration with a high uptake capacity and continuous-flow treatment capability of 2200 kg wastewater per kg sorbent, lowering Pb^2+^ levels below the WHO limit (10 ppb).

Deep eutectic solvent (DES) is a combination of two or more low-cost ingredients that can be mixed to form a uniform composition. An organic component that works as a hydrogen bond donor (HBD) and a compound or salt that operates as a hydrogen bond acceptor (HBA) are mixed to form a DES. The DES has a lower melting point than the individual constituents [30,31,32]. DESs are distinguished by their lack of flammability, low vapor pressure, and wide liquid range [33]. DESs have been utilized in different membrane applications due to their aforementioned qualities. DESs can modify MOFs and their combined incorporation into membranes increased the effectiveness of the membranes. The physical modification of the materials with DES has been extensively reported, which is why there was a need for chemical DES-mediation for a new trend to attract the attention of researchers; it provides an excellent medium for synthesis and opens pathways toward green solvent adoption to improve the sustainability of the process [34].

Although deep eutectic solvents (DESs) have been widely used in the past for the post-synthetic functionalization of materials, their function as chemical mediating agents during MOF formation to control the particle structure and interfacial properties has received no attention at all. In order to modify the structural properties and functionality of a MOF, a DES made of ethylene glycol and CTAB was added as a mediator during the synthesis process. The organic linker, pyridine-2,5-dicarboxylic acid, provided dual coordination via carboxylate and pyridinic nitrogen groups. In the synthesized DES-mediated MOF (DM-Zn-PDC@MOF), the presence of DES during MOF formation affected nucleation behavior and coordination interactions, leading to the improved structural stability, dispersion characteristics, and availability of accessible functional sites. To remove Pb(II) and Cr(III) ions from aqueous solutions, the produced DM-Zn-PDC@MOF was then added as a functional filler to polysulfone-based mixed matrix membranes (PSf/MMMs). It is anticipated that the DES-mediated chemical modification will increase the availability of active adsorption sites and improve heavy metal ion separation performance by promoting a uniform particle dispersion within the membrane matrix and enhancing MOF–polymer interfacial compatibility. Using a DES as a structure-directing mediator during MOF synthesis instead of a traditional physical modifier allows simultaneous control over MOF structural properties and membrane–filler interfacial interactions. This chemical combination has not been reported yet, which is the novelty of this work. The main objective is to increase the flux and heavy metal adsorption using the synergistic effects of DES-mediated MOFs, which will facilitate metal concentration for recovery and recycling. By enabling both water remediation and valuable metal resource reclamation through scalable and green chemistry-integrated membrane technology, this approach might be able to satisfy sustainability and circular economy goals through further processing.

## 2. Materials and Methods

### 2.1. Materials

The materials used in this study include cetyltrimethylammonium bromide (CTAB) (>99.0%) from BIO BASIC Inc., Markham, ON, Canada, ethylene glycol (≥99.0%), and zinc nitrate hexahydrate Zn(NO_3_)_2_. 6H_2_O (98%), pyridine-2,5-dicarboxylic acid (H2PDC:isocinchomeronic acid) (98%), polysulfone with av. MW ~22,000 (≥97%), ethanol (≥99.8%), and acetone (>99.5%) from Sigma–Aldrich, St. Louis, MO, USA, and N-methyl-2-pyrrolidone (NMP:99.5%) from RCI Labscan Limited, Bangkok, Thailand. Deionized (DI) water was obtained from a water purification unit (BOECO pure PLUS, Hamburg, Germany). All the reagents used were analytical grade.

### 2.2. Synthesis of DES

The DES was synthesized by mixing CTAB and ethylene glycol at a 1:0.5 ratio by mass for 3 h at 80 °C, in which CTAB was used as a hydrogen bond acceptor (HBA) and ethylene glycol was used as a hydrogen bond donor (HBD). The mixture was stirred in a round-bottom flask having three openings to attach a condenser and thermometer. A homogeneous mixture was synthesized, which solidified below 80 °C, and above this temperature, it was a liquid [35].

### 2.3. DES-Mediated MOF Synthesis

**Figure 1:**
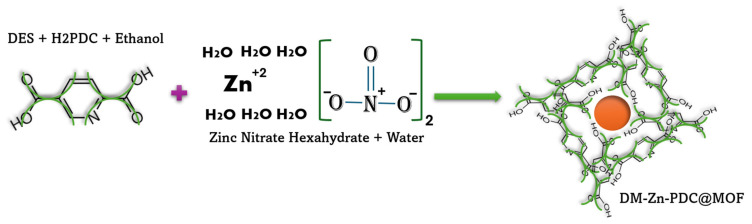
Synthesis of DM-Zn-PDC@MOF with organic and inorganic solutions using green solvents.

DM-Zn-PDC@MOF was synthesized by mixing 1.34 g of Zn(NO_3_)_2_.6H_2_O in 30 mL of DI water, which was an inorganic solution, and another mixture was formed by mixing 2.16 g of CTAB/EG-based DES and 0.52 g of pyridine-2,5-dicarboxylic acid (H2PDC) in 30 mL of ethanol, which was an organic solution. After 30 min, both solutions were poured into a Teflon-lined hydrothermal autoclave. The reaction was conducted at 120 °C for 12 h in a heating oven. Upon cooling to room temperature, DM-Zn-PDC@MOF was isolated by centrifugation at 5000 rpm for 8 min. The product was subsequently washed three times with ethanol and once with acetone. Afterward, the MOF was dried in an oven at room temperature for 24 h and stored in a dry environment [36]. Figure 1 shows the scheme of the synthesis.

### 2.4. Synthesis of Membranes

The membranes were synthesized using 5%, 10%, 15% and 20% of the synthesized MOF in a polysulfone matrix. The overall composition of each membrane is given in Table 1.

**Figure 2:**
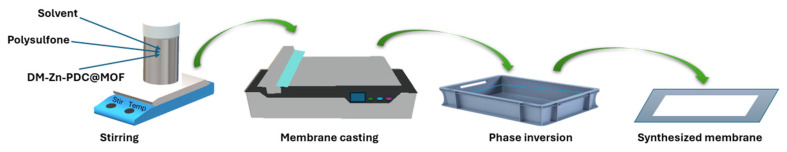
Synthesis of a membrane via phase inversion using a water bath.

Each membrane composition was stirred for 24 h in a closed vial at 400 rpm. After stirring, the blended mixture was poured on a glass slide and spread using a doctor’s blade with a 200 µm thickness in a membrane casting machine at room temperature. Then, the glass slide was completely dipped in the water bath to allow phase inversion of the solvent with water. After 30 min, the membrane was removed from the water bath and stored in a vessel in the form of a roll, which was dipped in the fresh water [37]. Figure 2 shows the scheme for the synthesis of the membranes developed in this study.

Afterwards, the water uptake, leaching ratio, and pure water flux were calculated. The relative hydrophilic–hydrophobic properties of the membranes were evaluated by measuring water uptake [37]. For this, a piece of the membrane with dimensions 2.5 × 2.5 cm was cut. After drying at 60 °C for 2 h, the weight of the dry sample (W_dry_) was obtained using a weight balance, and after weighing, the sample was placed in DI water overnight; then, after wiping the external surface with a dry tissue, the weight of the wet sample (W_wet_) was obtained. Then, the percentage of the water uptake was determined using the following equation [38]:

$$Water\;uptake\;\left(\%\right)=\frac{W_{wet}-W_{dry}}{W_{dry}}\times 100$$

Leaching may be associated with the partial removal of loosely bound filler particles and residual solvent from the PSf matrix due to minor inconsistencies in filler dispersion within the membrane structure. The 2.5 × 2.5 cm piece of membrane was dried for 2 h at 60 °C, weighed (W_1_), and then immersed in water. The water was replaced with fresh water on a daily basis. After one week, the membrane was gently wiped with tissue paper, dried for 2 h at 60 °C, and weighed (W_2_) again. The following equation was used to evaluate the leaching ratio [39]:

$$Leaching\;ratio\;\left(\%\right)=\frac{W_{1}-W_{2}}{W_{1}}\times 100$$

Many membranes were synthesized, and the best membranes, based on their uniform dispersion and opaque nature, were selected to be tested, as the membranes having clear tiny holes, due to the presence of bubbles in the doping solutions, were not suitable to be tested. The pure water flux, using DI water, through the membranes, was investigated once, but the rejection of heavy metals (using 150 ppm sol.) was examined three times to take an average using a dead-end filtration setup with a 300 mL capacity. Cyclic filtration using a cross-flow setup was not performed in this study, but it can open a way toward future work to determine the long-term stability and durability. To stabilize the system with respect to flow and pressure, it was run for 15 min so that the empty spaces in the module would be filled. At room temperature, filtration was performed using an active membrane surface area of 14.6 cm^2^ with an applied pressure of 8 bar via compressed air. The following equation was used to determine the flux [37]:

$$Flux=\frac{v}{A\times t}$$

where v, A, and t are the volume of permeate (l), the active surface area of the membrane sample (m^2^), and the time of permeation (h).

The Grout Elford Ferry equation was the basis of the calculations of pore size and porosity [40]:

$$d_{p}=2\times \sqrt{\frac{8µlQ(2.9-1.75\varepsilon )}{\varepsilon A∆P}}$$

where $d_{p}$, µ, l,Q,A, ∆P, and ε are pore diameter (pore size = nm), viscosity of water at room temperature, thickness of the membrane (m), water flux (m^3^·s^−1^), active surface area of membrane (m^2^), applied pressure (Pa), and the porosity of membrane, respectively. The porosity was calculated using the following equation:

$$\varepsilon =\frac{{W_{wet}-W}_{dry}}{Vd}$$

where W_wet_, W_dry_, V, and d are weights (g) of wet membrane sample, dry membrane sample, volume of water (mL), and density of water (g·cm^−3^), respectively.

## 3. Characterization of DM-Zn-PDC@MOF and the Membranes

The materials used and synthesized in this research were characterized by Fourier transform infrared spectroscopy (FTIR), thermogravimetric analysis (TGA), atomic force microscopy (AFM), X-ray diffraction (XRD), scanning electron microscopy (SEM)/Energy dispersive X-rays (EDX), and contact angle (CA) measurements. A PerkinElmer Spectrum 100 FTIR instrument (PerkinElmer, Rodgau, Germany) was utilized to detect the functional groups of the materials and membranes, and to detect whether the membrane synthesis system underwent a physical change or a chemical reaction. TGA was conducted using a PerkinElmer instrument (Model, STA 8000, PerkinElmer) to investigate the thermal stability of the materials and membranes. A sample weight of 20 mg was placed in the oven at 60 °C for 1 h for initial drying to evaporate absorbed water vapors. After drying, the samples were analyzed in a nitrogen environment from 25 °C to 570 °C with a temperature changing rate of 12 °C per minute. AFM (Nanosurf C3000, Nanosurf, Liestal, Switzerland) was utilized, and the analysis was done using a silicon tip of 10 nm, a resonance frequency of 70 kHz, and a noncontact tap-spring of 2 Nm^−1^ to observe the surface morphology of membranes. XRD patterns of the materials and membranes were obtained using an AXRD-LPD system with a 50 Hz frequency, 20 A current, and 220 V voltage to determine the structures. SEM and EDX detailing were done with a Zeiss microscope (Model, EVO 15, Zeiss, Jena, Germany) with a 12 kV accelerating voltage; the samples were prepared by breaking the membranes in liquid nitrogen to fix the structural morphology, drying the pieces, and applying a sputter coater to coat the samples with gold. Contact angle measurements were done using a goniometer (OCA20, Dataphysics Instruments, Filderstadt, Germany) to determine the water contact angle using a 2 µL droplet size, from which the hydrophilic properties of dried membranes were derived.

## 4. Results and Discussion

### 4.1. FTIR Spectroscopy

**Figure 3:**
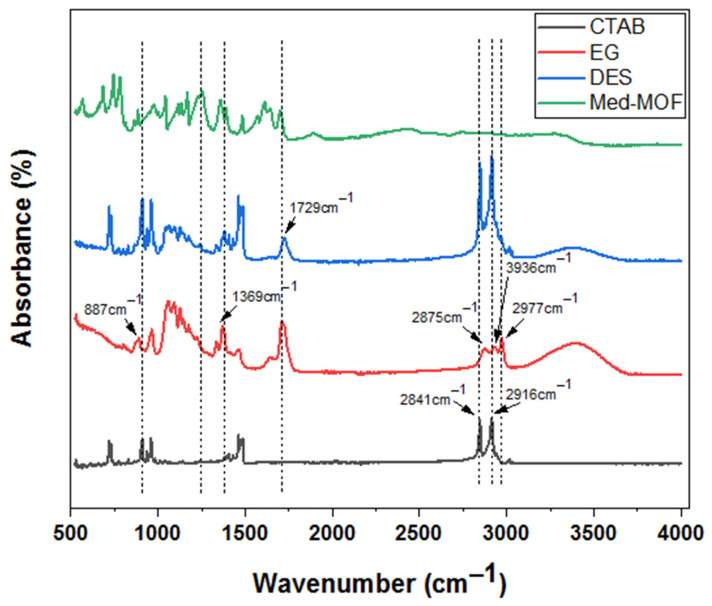
FTIR spectra of CTAB, ethylene glycol, DES, and DM-Zn-PDC@MOF.

FTIR spectra of DES, CTAB, ethylene glycol, and the synthesized DM-Zn-PDC@MOF are displayed in Figure 3. The C–H bond stretching vibration from CTAB is represented by the distinctive peaks seen at 2922 cm^−1^ and 2850 cm^−1^ [41]. The presence of hydroxyl groups is confirmed by the broad peaks found at approximately 3391 cm^−1^, which correspond to O-H stretching vibrations [42]. C-H stretching vibrations were identified because of the peaks at 2800–3000 cm^−1^ [43], alongside characteristic C-O stretching vibrations at 1000–1300 cm^−1^ [44] for the ethylene glycol moiety. The presence of hydroxyl groups in DES was confirmed by the broad peaks around 3292–3293 cm^−1^, which correspond to the O–H stretching vibrations [45]. Peaks in the 500–900 cm^−1^ range show wagging vibrations linked to the O-H group of ethylene glycol [46]. Furthermore, C-H stretching vibrations were identified because of peaks at 2935–2936 cm^−1^ and 2878–2880 cm^−1^ [47], alongside characteristic C-O stretching vibrations [48], confirming that the anticipated molecular structures are present. The individual chemical structures were confirmed to have remained intact after DES formation by the similarity between the DES spectra and the reference spectra of the pure constituents. This result shows successful integration devoid of chemical deterioration [49].

The influence of H2PDC on the final MOF structure is responsible for the disappearance of peaks in the DM-Zn-PDC@MOF spectrum at 3098–2858 cm^−1^. On the other hand, this decrease in intensity in comparison to pure CTAB might suggest that surfactant molecules are either partially integrated into the framework structure or trapped as templating agents inside the MOF pores [50]. The C–H stretching vibration of the pyridine ring is represented by the distinctive band at 3421 cm^−1^. Furthermore, the carboxyl group in tension and bending vibration is represented by the characteristic band at 1617 cm^−1^. The C-O vibrations are attributed to the weak band at 1283 cm^−1^ [51]. Coordinated ethylene glycol is present as a co-ligand in the 1000–1300 cm^−1^ range, which may help to bridge connections between zinc nodes. The overall spectral profile of DM-Zn-PDC@MOF demonstrates that a combination of primary coordination bonds between zinc and H2PDC, secondary coordination involving ethylene glycol, and structural templating by CTAB molecules has resulted in successful framework formation.

**Figure 4:**
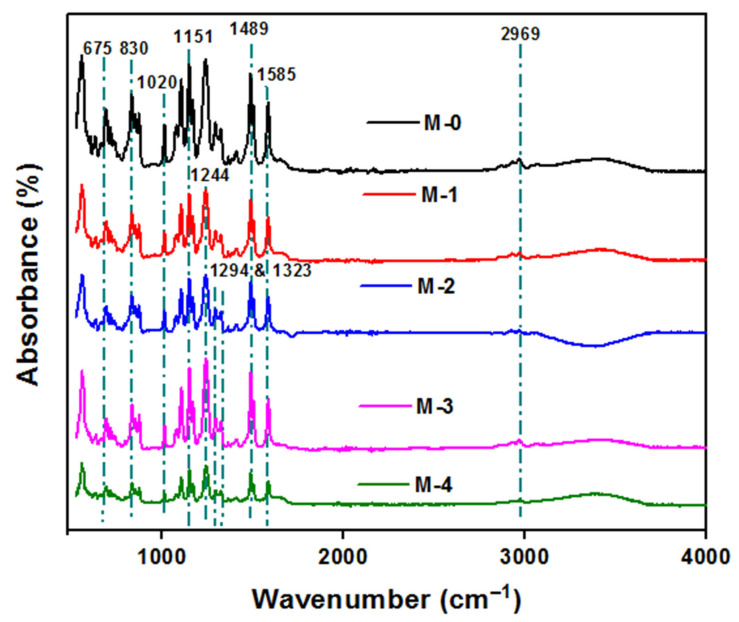
FTIR spectra of MMMs.

The bonding chemistry of pristine PSf and DM-Zn-PDC@MOF-loaded polysulfone membranes was examined using FTIR analysis, as shown in Figure 4. Several bands of absorption were detected in the spectrum of the pristine PSf membrane: C–C aromatic bonds at 1585 cm^−1^, C–O–C stretching at 1244 cm^−1^, and O–S–O stretching at 1151 cm^−1^. These bands are characteristics of the sulfone group. At 830 cm^−1^ and 1020 cm^−1^, C–H stretching of the aromatic ring was also observed. The ether (C–O–C) stretching of the PSf moiety was identified as the source of a strong characteristic peak at 1243 cm^−1^, while other stretching vibrations of C–H displayed an absorption band at 2969 cm^−1^. Additionally, aromatic carbon–carbon (C=C) bond stretching vibrations were detected at 1488.35 cm^−1^, 1504.03 cm^−1^, and 1585.98 cm^−1^. The asymmetric stretching of the O–S–O group is indicated by the appearance of peaks at 1169.77 cm^−1^, 1294.79 cm^−1^, and 1323.99 cm^−1^. These FTIR spectral values are consistent with the results that were reported by Zaman et al. [39]. All DM-Zn-PDC@MOF-loaded polymeric membranes experience filler layering rather than new bond formation, resulting in only peak suppressings.

### 4.2. Thermogravimetric Analysis (TGA)

**Figure 5:**
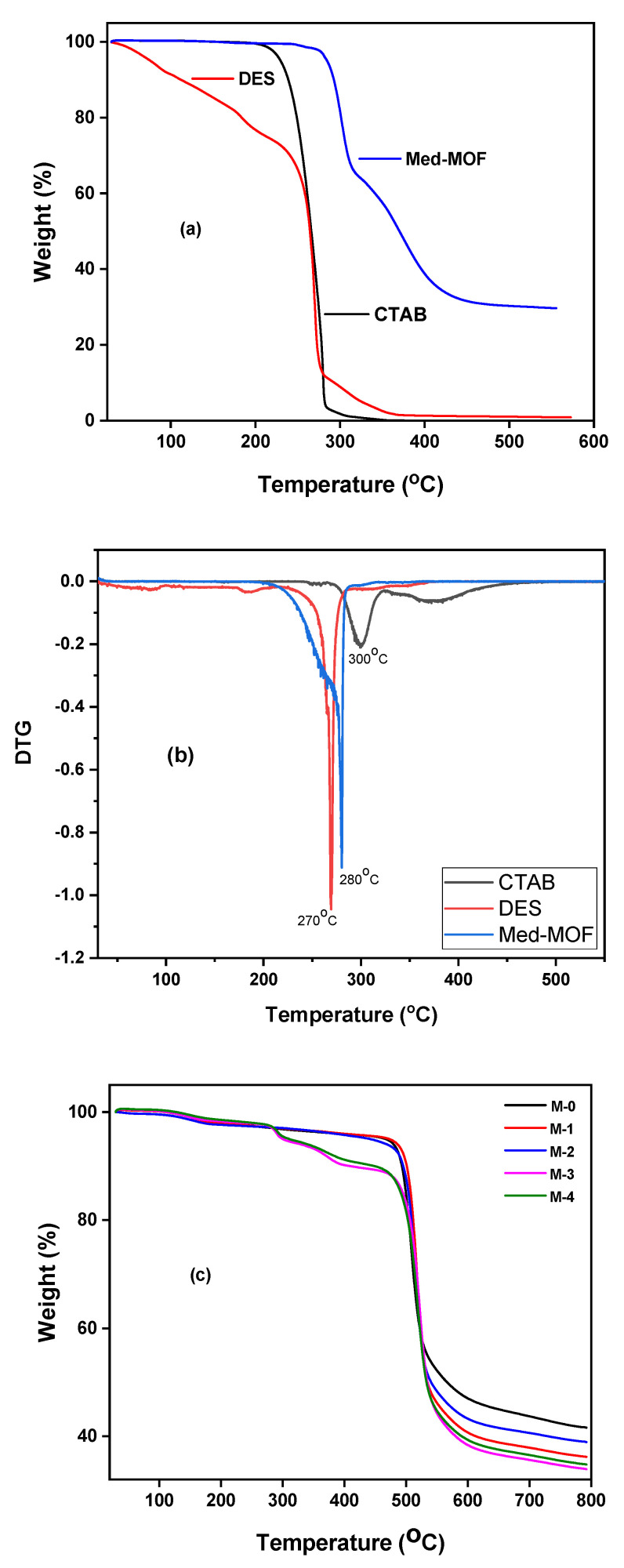

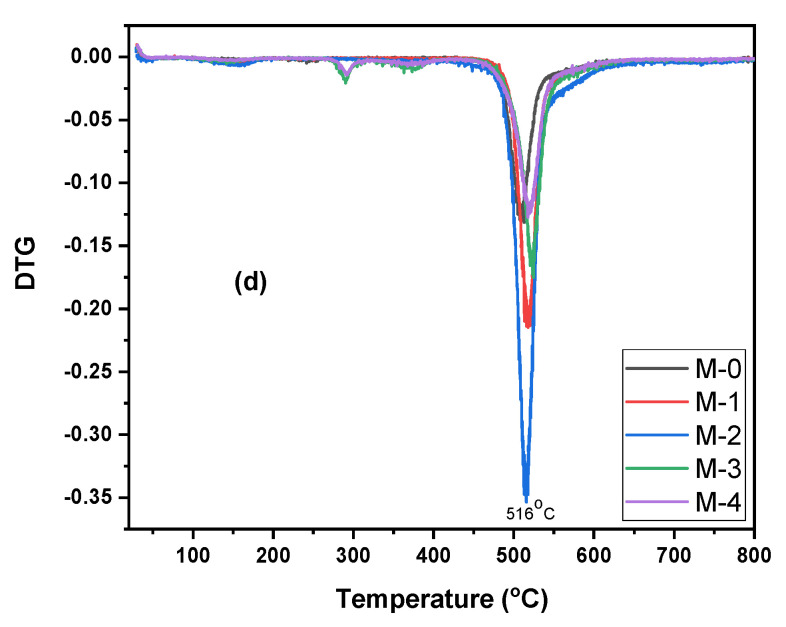
(**a**) TGA of the materials and (**b**) DTG of the materials. (**c**) TGA of the membranes and (**d**) DTG of the membranes.

Figure 5a,b depict the results of the thermogravimetric analysis (TGA) and differential thermogravimetry (DTG) of the pure DES, CTAB, and the resultant DM-Zn-PDC@MOF. The thermal stability of these materials was tested at 30–550 °C. The initial weight losses refer to the evolution of the remaining absorbed vapors. In the curve for DES, the gradual weight loss around 200 °C is attributed to the loss of ethylene glycol vapors, which acts as one of the prime components in the synthesis of DES. The boiling temperature of ethylene glycol lies in the range of 197–198 °C, and after exceeding that limit, the molecules tend to undergo thermal decomposition and contribute to the downfall of the curve from the weight axis (ordinate). The significant weight loss observed in the curves of DES and CTAB, both near 220–300 °C, refers to the chemical decomposition of CTAB chains. Overall, at 260–400 °C, major weight loss was observed. The DM-Zn-PDC@MOF was observed to be a highly stable material that can be used for high-temperature applications.

Figure 5c,d show the results of the thermogravimetric analysis and differential thermogravimetry of the synthesized membranes. The analysis was done under the same conditions using the same weight of membranes from 25 °C to 790 °C. Because of the aromatic rings of the polysulfone, the membranes were stable up to 500 °C. The initial 8–10% loss of weight indicated the shrinkage of membranes due to the evolution of the residual water and the solvent stuck in the pores of the membrane. The sudden change of 30–40% at temperatures above 500 °C indicated the breakage of the bonds of the polymer matrix and the filler inside, as this temperature is higher than the boiling point of PSf and the maximum stable temperature of the DM-Zn-PDC@MOF filler. The small changes presented in the different colors also show the change in the amount of the filler in the polymer matrix. These trends are similar to those reported in previous studies [52,53]. 

### 4.3. Atomic Force Microscopy (AFM)

**Figure 6:**
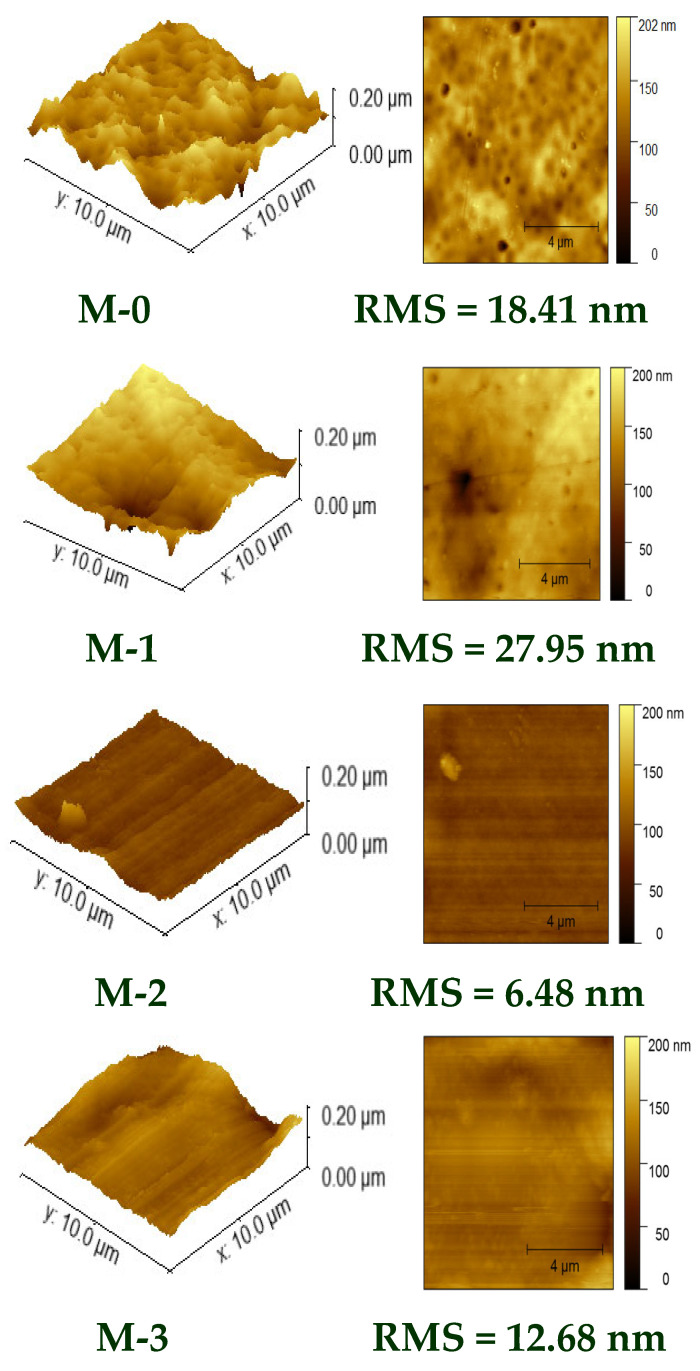

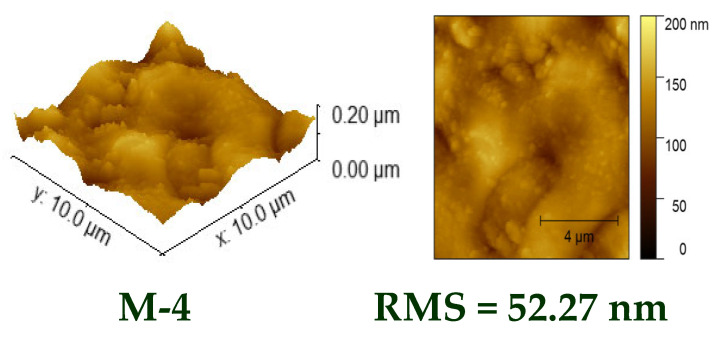
Atomic force microscopy images of the pristine (M-0) and filler-loaded membrane samples.

The impact of MOF loading on the surface roughness of membranes was demonstrated by AFM analysis, and the results are shown in Figure 6. Due to the small amount of MOF added, the roughness of M-1 increased compared to the pristine membrane M-0. During phase inversion, a few MOF crystallites might preferentially migrate toward the membrane surface because the polymer chains cannot evenly distribute them throughout the bulk at such a low concentration. Particles remain as isolated protrusions or clusters at the top layer because there are insufficient particles to create particle–particle interactions and stable embedding within the matrix. The higher RMS roughness observed for M-1 is caused by these poorly anchored surface features, which break the otherwise smooth polymer continuity [54]. On the other hand, the RMS roughness reaches its lowest value when the DM-Zn-PDC@MOF loading reaches 10%. This reduction may result from the templating effect of DES during synthesis, which produces more uniformly faceted, polymer-compatible crystallites that are easier to embed and align within the membrane matrix, in addition to the intrinsic particle geometry and surface chemistry of DM-Zn-PDC@MOF. The polymer matrix reaches its saturation limit for efficiently dispersing MOF particles at higher loading (M-4). Beyond this point, polymer–filler interactions are weakened because the available polymer chains are unable to completely wet and encapsulate all crystallite surfaces.

Consequently, during the phase inversion process, the MOF particles interact with one another more and more, stimulating particle–particle clustering and surface migration. Additionally, the increased solid fraction speeds up the exchange of solvents and non-solvents, destabilizing the casting front and pushing aggregates toward the top surface, thereby intensifying topographic heterogeneities. Phase separation induced particle accumulation at the skin layer, extensive agglomeration, and limited polymer wrapping, all of which worked together and explain why the surface roughness in M-4 rises quickly rather than gradually. This is the reason that M-4 exhibits a higher permeability and pure water flux than the other membranes.

### 4.4. X-Ray Diffraction (XRD)

**Figure 7:**
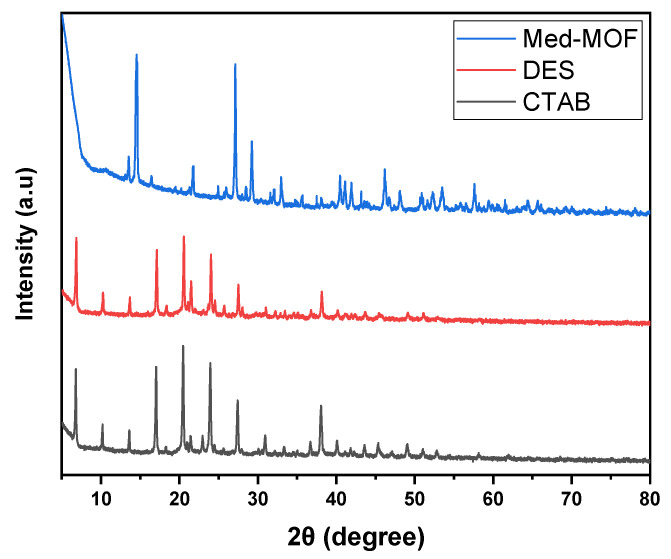
XRD patterns of CTAB, DES, and DM-Zn-PDC@MOF.

The XRD pattern confirmed the phase purity and crystalline nature of DM-Zn-PDC@MOF, as shown in Figure 7. The DM-Zn-PDC@MOF diffraction pattern displays the same collection of distinctive peaks as those previously documented by Mao et al. [51], confirming that the DES-assisted method was effective at obtaining the basic framework topology. Interestingly, DM-Zn-PDC@MOF pattern lacks the strong reflections associated with pure CTAB/EG-based DES and CTAB, indicating that these substances do not remain as distinct crystalline phases following synthesis reaction and washing. Compared to what Mao et al. reported [51], DM-Zn-PDC@MOF exhibits minor changes in relative intensities and peak positions. The impact of the CTAB/EG-based DES during nucleation and growth is responsible for these variations. Specifically, during the early phases of assembly, the robust hydrogen-bonding network of the DES probably interacts with Zn^+2^ centers and the H2PDC ligand to direct the crystallization process. The observed peak variations are caused by a templating or structure-directing effect that modified the orientation of crystal growth and introduced minute modifications to the unit cell parameters. Despite the minor changes caused by the DES environment, the final DM-Zn-PDC@MOF is phase-pure, crystalline, and structurally consistent. XRD spectra of the membranes can be seen in Figure S1. There are no sharp peaks; the reason is the low signal-to-noise ratio because a thin membrane film of each sample was analyzed. As the polysulfone is amorphous and the filler has been used in a very small amount compared to the polymer, and because it is encapsulated into the polymer matrix, the crystalline property of DM-Zn-PDC@MOF was suppressed, and the polymer properties became dominant in the analysis.

### 4.5. SEM and EDX

**Figure 8:**
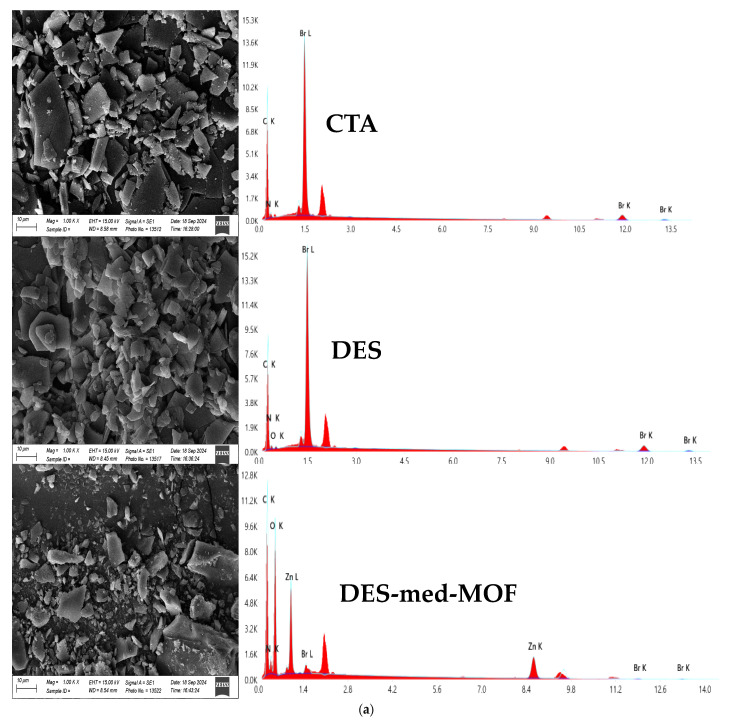

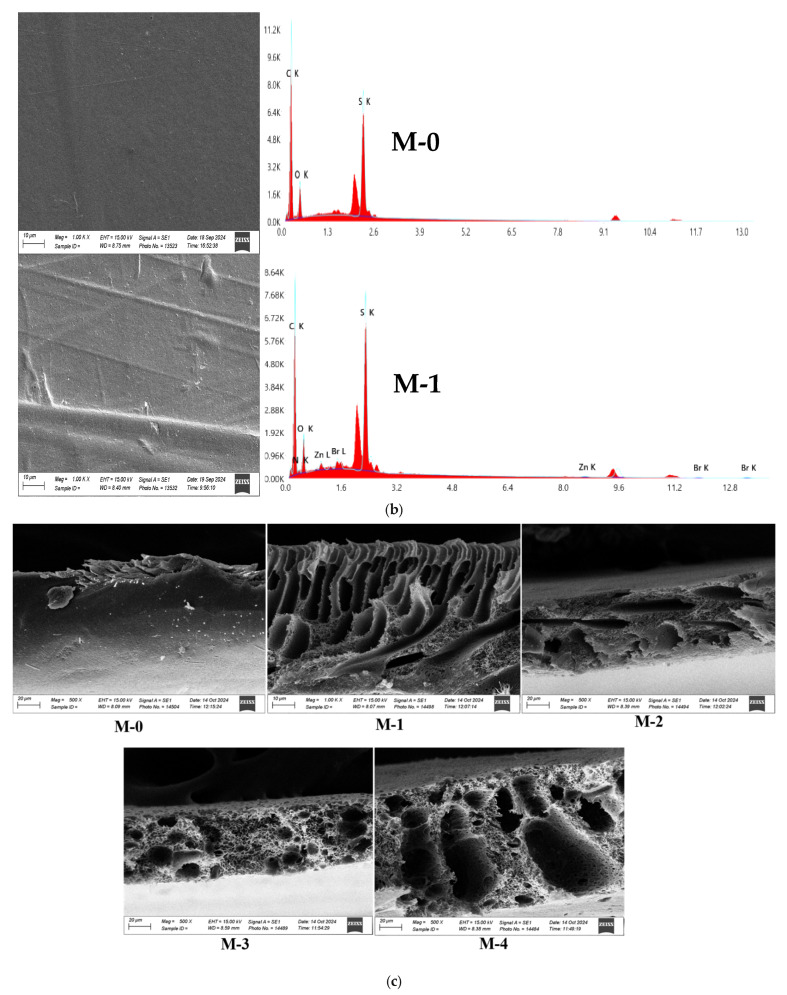
(**a**) SEM and EDX analyses of the materials. (**b**) SEM and EDX analyses of the membranes. (**c**) SEM images of the cross-sections of the membranes.

Figure 8a,b show the surface morphology of the materials and the membranes with their elemental analyses using Energy Dispersive X-rays (EDX). The particles of all the materials are uniform and also uniformly dispersed in the PSf matrix. The particle size differed due to the agglomeration of the particles. The crystals of the materials in the image can be observed clearly. The elemental analysis of CTAB, DES, and DM-Zn-PDC@MOF confirms the presence of their elements and that CTAB has no oxygen atom, while DES has one oxygen atom because of the ethylene glycol component. Furthermore, only DM-Zn-PDC@MOF has a Zn atom that is not part of CTAB or DES. In line with the FTIR analysis, this result also confirms the success of the synthesis of DM-Zn-PDC@MOF. The pristine M-0 membrane is only showing the elements of the polysulfone, but M-1 is showing the extra Br and Zn atoms that prove the uniform distribution of the DM-Zn-PDC@MOF in the membrane. The membrane composition with a minimal MOF percentage (5 wt%), M-1, confirms the MOF distribution and results of the elemental analysis; hence, the higher MOF percentages in M-2 to M-4 will also do so.

The DES mediation has a significant influence on regulating both the MOF particle organization and the subsequent development of the membrane pore architecture, as evidenced by the SEM cross-sectional analysis of DM-Zn-PDC@MOF-loaded membranes. As the baseline morphology of the unmodified polymer matrix without any filler features, the pristine PSf membrane (M-0) has a smooth surface appearance, minimal porosity, and a relatively dense and uniform cross-sectional structure. When DM-Zn-PDC@MOF is added to M-1 at low loading, the morphological landscape changed dramatically. This is because the DES-templated MOF particles act as nucleation sites during phase inversion, resulting in the development of unique finger-like pore structures that extend from the top surface into the membrane cross-section [55]. The functional groups from CTAB first form discrete MOF domains that orient with their hydrophobic alkyl chains, influencing local polymer–solvent interactions and pore formation patterns. M-2 exhibits a more intricate porous structure with a mix of finger-like macrovoids and intermediate-sized pores dispersed across the cross-section. This suggests that a higher DM-Zn-PDC@MOF loading intensifies the effect, in which the components of MOF start to form networks that affect the formation and stability of pore walls and lead the system toward particle organization. The critical templating threshold where the DM-Zn-PDC@MOF system attains a perfect balance is represented by the cross-sectional morphology of M-3, which displays the most prominent and ideal macrovoid structure with large, well-defined spherical and elliptical pores dispersed throughout the membrane thickness. At the same time, a sufficiently high MOF concentration generates a percolation network in which the hydrophilic carboxylate and pyridine functionalities of the H2PDC ligands of DM-Zn-PDC@MOF are completely incorporated into the pore architecture. The higher loading of DM-Zn-PDC@MOF results in particle agglomeration and uncontrolled void formation that overwhelms and leads to the templating-directed pore development mechanism. In contrast, M-4 displays extremely large macrovoids, with some pores approaching the full membrane thickness, along with signs of structural compromise such as pore coalescence and irregular pore shapes. The DM-Zn-PDC@MOF system shifts from hydrophobic-dominated templating at lower loadings to hydrophilic MOF-functionalized templating at the optimal loading (M-3) and finally results in a bimodal templating effect that achieves maximum morphological control. Excessive loading (M-4) caused templating system failure and the loss of the controlled pore architecture development. The cross-sectional images of the membranes can be seen in Figure 8c.

### 4.6. Contact Angle Measurements

**Figure 9:**
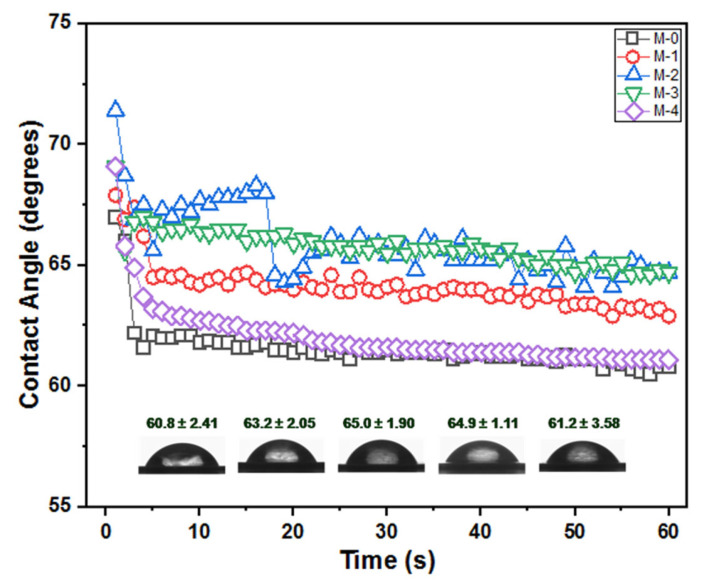
Variations in the contact angles of pristine and filler-loaded membrane samples as a function of time.

Through a complex interaction between DES templating mechanisms and MOF particle organization, the contact angle analysis offers strong evidence for the unique templating role of DM-Zn-PDC@MOF in regulating membrane surface properties, as shown in Figure 9. When DM-Zn-PDC@MOF is systematically added in samples M-1 to M-4, the result is a complex templating-driven surface evolution that is directly correlated with the DM-Zn-PDC@MOF particle morphology and distribution. The pristine membrane (M-0) displays a baseline contact angle of 60.8 ± 2.4°, indicating the inherent hydrophilicity of the unmodified polymer matrix. The reason for the increased contact angles at lower MOF loadings (M-1, 63.2 ± 2.1°; M-2, 65.0 ± 1.9°; and M-3, 64.9 ± 1.1°) is that the DES templating effect produces distinct, well-organized MOF particles that preferentially orient their hydrophobic alkyl chains toward the surface. This effectively creates hydrophobic microdomains that momentarily conceal the underlying hydrophilic polymer and MOF functional groups. This phenomenon is indicative of the dual nature of the DM-Zn-PDC@MOF system, in which the cationic surfactant component functions as a structure-directing agent by virtue of its amphiphilic nature, and influences surface orientation in addition to controlling MOF crystal growth. A templating threshold effect is demonstrated by the critical transition observed in sample M-4, which surpasses even the pristine membrane in hydrophilicity and achieves the lowest contact angle of 61.2 ± 3.6°. This occurs through two synergistic mechanisms. First, the DM-Zn-PDC@MOF begins to dominate surface chemistry by exposing its inherent hydroxyl groups and facilitating hydrogen bonding networks with water molecules. Second, the high MOF concentration creates a percolation network where the hydrophilic carboxylate and pyridine functionalities from H2PDC ligands become the dominant surface features. The DM-Zn-PDC@MOF templating system switches from a hydrophobic CTAB functional group-dominated surface organization to hydrophilic EG functional groups and MOF-functionalized surface exposure at this ideal loading, resulting in a bimodal templating effect that optimizes water affinity. The DES has produced well-integrated, structurally stable MOF particles that retain their templated surface orientation without a rapid reorganization. Moreover, the temporal stability of contact angles across all samples over a 60 s measurement period was observed. Both of these factors supported the overall synthesis, as there was no significant difference in the contact angles of all the membranes. This confirms that the DM-Zn-PDC@MOF synthetic strategy offers precise control over the membrane surface properties through templating-directed MOF incorporation and surface functionalization.

## 5. Pore Profile

### 5.1. Water Uptake

**Figure 10:**
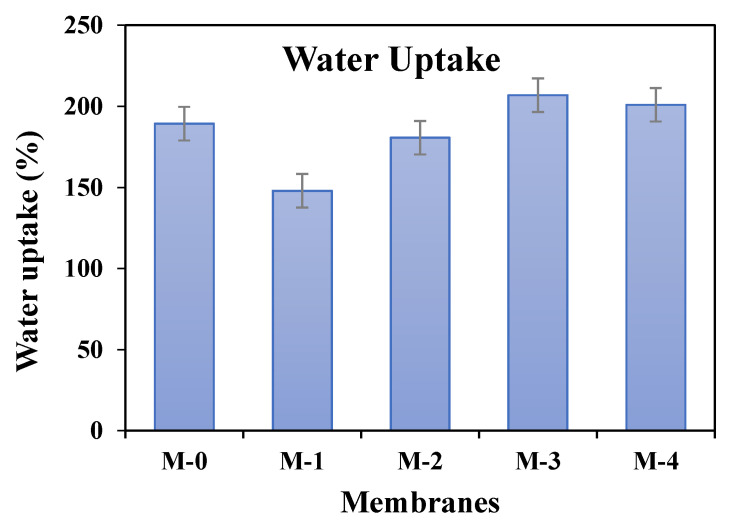
Water uptake (%) by pristine (M-0) and filler-loaded membrane samples (M-1 to M-4, n = 3, and S.D ≤ 10%).

Measurements of water absorption (%) correlated with SEM cross-sectional morphology yield insights into the structure–property relationships of DM-Zn-PDC@MOF-loaded membranes. Figure 10 reveals that the pristine membrane M-0 reflects a baseline water absorption of approximately 190 ± 9%, which correlates with its dense, compact cross-sectional structure observed in the SEM analysis, where the limited porosity restricts water penetration and storage within the membrane matrix. The formation of finger-like macrovoid structures, which function as transport pathways rather than water storage regions and restrict water penetration in the absence of external pressure, is responsible for the decreased water absorption observed for M-1 (≈150 ± 10%). Furthermore, the addition of DES-mediated DM-Zn-PDC@MOF enhances polymer–filler interfacial compatibility, decreases interfacial voids and free volume within the PSf matrix, and further reduces water uptake by the membrane due to the presence of hydrophobic alkyl chains derived from the functional groups of CTAB. The observed increase in water absorption (≈185 ± 8%) is consistent with improved interconnected porosity with intermediate-sized pores in the SEM cross-section of M-2. This suggests that the incorporation of DM-Zn-PDC@MOF successfully produces pore architectures that increase the internal surface area for water interaction and retention. With large spherical and elliptical pores that provide the maximum internal volume while maintaining structural integrity, M-3 has the most noticeable and well-defined macrovoid structures. This results in the highest water absorption (≈210 ± 8%). This implies that M-3 has an ideal combination of mechanical stability and porosity. On the other hand, M-4 exhibits large macrovoids with indications of structural compromise and pore coalescence, which accounts for the minor decrease in water absorption (≈200 ± 10%) compared to M-3. These observations indicate a critical threshold effect, whereby optimal DM-Zn-PDC@MOF loading maximizes water uptake through an ideal pore architecture, whereas excessive loading leads to structural instability that reduces water retention despite the minor increased porosity.

### 5.2. Leaching Ratio

**Figure 11:**
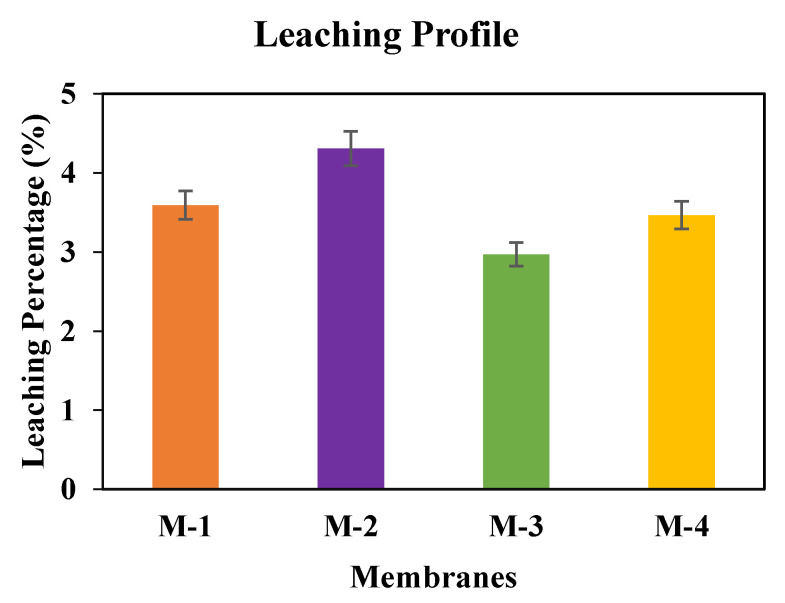
Leaching ratio of DM-Zn-PDC@MOF through membranes (n = 3, and S.D ≤ 5%).

The leaching analysis shown in Figure 11 demonstrates the structural integrity and filler retention capabilities of the DM-Zn-PDC@MOF-loaded membranes, highlighting the essential role of DM-Zn-PDC@MOF–polymer matrix interactions. The pristine membrane (M-0) showed no detectable leaching, establishing a baseline for the stability of filler-free membranes and confirming the structural integrity of the polymer matrix [55]. The incorporation of filler via DES-mediated synthesis exhibits a systematic leaching pattern across various loadings. M-1 shows approximately 3.8% leaching, which can be ascribed to the initial incorporation phase, where a limited number of MOF particles may exhibit weak interactions with the polymer matrix, despite the influence of DM-Zn-PDC@MOF templating. The leaching percentage reaches a maximum of approximately 4.5% in M-2, suggesting that this loading signifies a critical threshold at which the DM-Zn-PDC@MOF particles start to compromise the retention capacity of the polymer matrix. This may be attributed to the incomplete integration of larger MOF aggregates formed via the internal functional groups. M-3 demonstrates the lowest leaching percentage at approximately 3.0%, indicating optimal compatibility between the MOF and polymer achieved via DES-mediated synthesis. The functional groups of the DES enhance MOF dispersion and foster interfacial adhesion through hydrogen bonding networks between the MOF particles and polymer chains. The leaching behavior in M-4 (approximately 3.6%) indicates that the higher MOF loading is maintained effectively, with a slight increase observed compared to M-3. This suggests that the DM-Zn-PDC@MOF system achieves its optimal efficiency at moderate loadings. Leaching may result from the removal of loosely bound DES components, unreacted precursors, or weakly integrated MOF particles that were not completely incorporated into the polymer matrix during membrane synthesis. All membrane samples exhibited acceptable leaching percentages that were significantly below 4.6% [37], indicating that the DES-mediated MOF incorporation strategy effectively produces stable MMMs for filtration applications. The DM-Zn-PDC@MOF system facilitates effective templating and integration mechanisms, preserving structural integrity while allowing for controlled MOF loading.

### 5.3. Pure Water Flux

**Figure 12:**
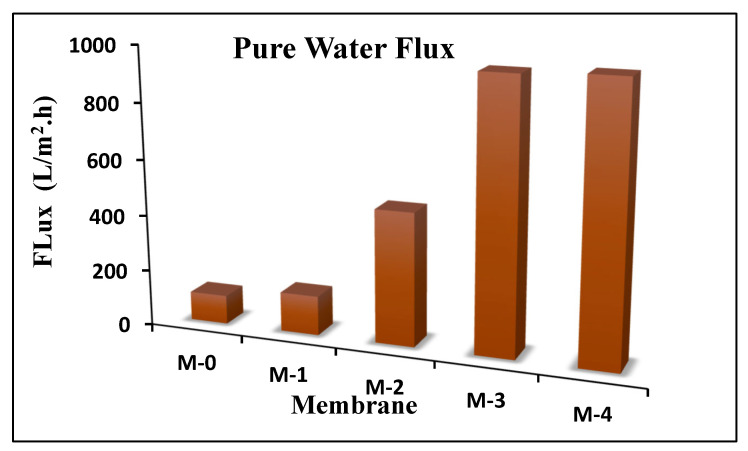
Pure water permeation flux of membranes.

Figure 12 shows the increasing trend of the flux with the increase in the amount of the DM-Zn-PDC@MOF filler, which refers to the nano-sized pores in the crystals of the filler and an increase in the pore size of membranes. Overall, the flux of membrane M-4 was increased by a factor of nine compared to M-0. There was no major difference in the flux of membranes M-3 and M-4 because of the agglomeration of the particles at higher concentrations [56]. The permeability of M-4 was the highest, which completely supports the findings of the characterizations discussed above.

### 5.4. Pore Size and Porosity

**Figure 13:**
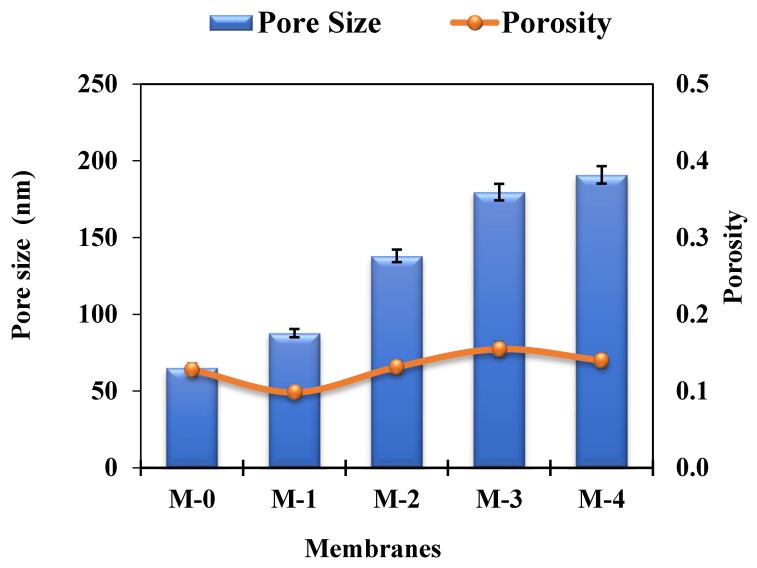
Pore size and porosity of membranes (n = 3, and S.D ≤ 5%).

The pore size and porosity are critical factors and significantly influence the interpretation of the data and are essential for understanding the pore structure of the membrane. Figure 13 shows the comparison of porosity and pore size of the pristine membrane with DM-Zn-PDC@MOF-based membranes. The porosity of M-0 was 0.128, and the maximum porosity was observed for M-3 (0.157), this refers to the fact that DM-Zn-PDC@MOF is intrinsically porous and creates additional channels for transport. However, the overall trend of porosity was different as it decreased from M-0 to M-1 and from M-3 to M-4. The two possible reasons for the observed porosity trends are polysulfone chain rigidification and agglomeration of the MOF particles at higher concentrations. The polymer chain rigidification results in tight chain packing near the filler surface. The pore size of the membrane increased from 65.2 nm to 190.9 nm. The pore size increased with the increase in filler loading. The rejection of the heavy metal ions cannot be explained by a steric hindrance mechanism because the pore size range of 65.2–190.9 nm is within the microfiltration regime and is much larger than the hydrated ionic diameters of Pb^2+^ and Cr^3+^ species, i.e., 8.02 Å [57] and 3.9 Å [58]. Rather, adsorption-assisted mechanisms primarily control the removal process. Strong inner-sphere complexation with carboxylate groups, additional interactions with pyridinic nitrogen sites, as well as hindrance from its larger ionic radius are the main factors controlling the removal of heavy metals. Additionally, without sacrificing removal efficiency, the larger pore size improves water permeability and allows for better ion mass transfer toward the active adsorption sites. As a result, rather than acting as a strictly size-selective barrier, the membrane serves as an adsorption-assisted microfiltration system where affinity-based interactions are primarily responsible for the removal of Pb(II) and Cr(III) ions.

### 5.5. Removal of Heavy Metals

**Figure 14:**
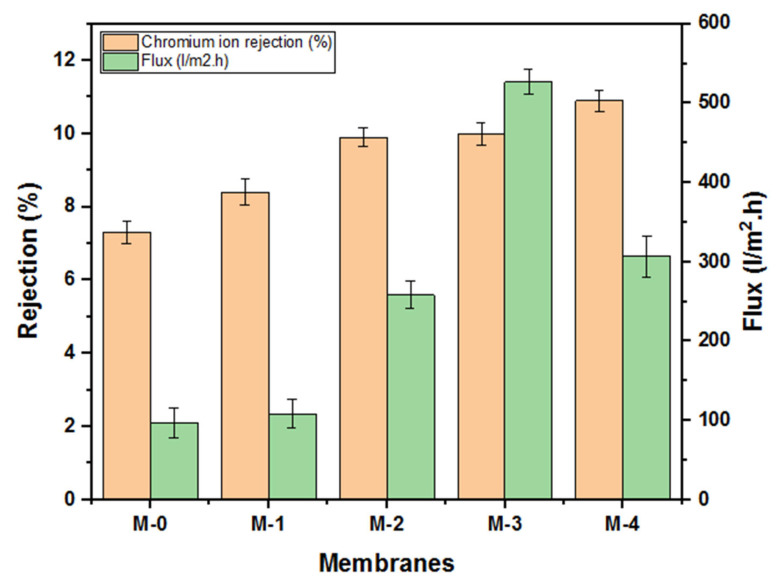
Cr(III) ion rejection and flux through the membranes (n = 3, and S.D ≤ 5% for rejection).

Standard solutions of chromium nitrate and lead nitrate were passed through the membranes to evaluate the removal of Cr(III) and Pb(II) ions from water. The initial concentrations of the Cr(III) and Pb(II) solutions were 150 ppm. The performance was tested three times, and the average was reported. Figure 14 shows the comparison of Cr^+3^ ion rejection with the flux. The overall rejection was not significantly higher, but it increased from M-0 to M-4. Strong inner-sphere complexation with carboxylate groups and additional interaction with pyridinic nitrogen sites are the main factors controlling the removal of Cr^3+^ ions. On the other hand, improved hydrophilicity and the creation of linked transport pathways caused the flux to increase from M-0 to M-3. Increased tortuosity, partial pore blockage, and potential particle agglomeration, which lower effective porosity, are responsible for a decrease in flux at a higher loading (M-4). Despite this, rejection kept improving, which is in line with the membrane’s increased adsorption capacity [59].

**Figure 15:**
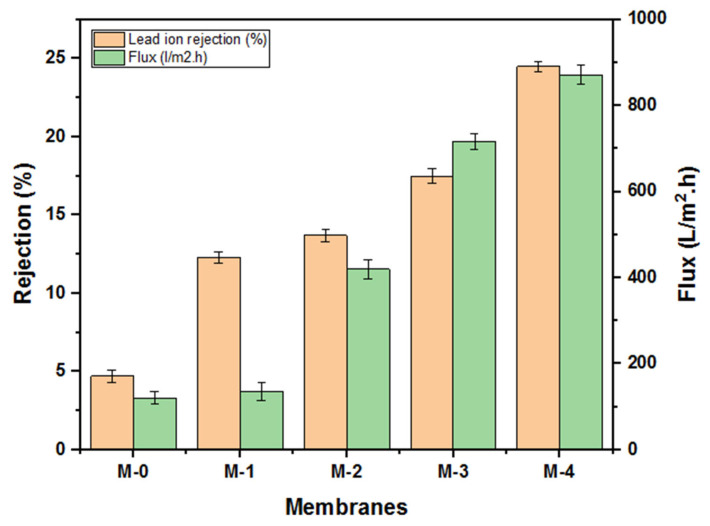
Pb(II) ion rejection and flux through the membranes (n = 3, and S.D ≤ 5% for rejection).

The rejection of Pb^2+^ ions was greater than that of Cr^3+^ ions and increased gradually from M-0 to M-4, peaking at 24.5% for M-4, as shown in Figure 15. Stronger coordination between Pb^2+^ and carboxylate (-COO^−^) groups, as well as hindrance from its larger ionic radius, are responsible for this improvement. Because of improved transport pathways and increased hydrophilicity, the flux also increased significantly with filler loading, from 120.6 to 871.8 L·m^−2^·h^−1^. Strong filler–polymer interactions reduced non-selective voids and encouraged efficient adsorption, pushing selectivity despite the high flux. Coordination, diffusion resistance, and multi-step adsorption all worked together to increase rejection, with M-4 exhibiting the best overall performance, in line with the findings from the characterization.

The comparison of the rejection of Cr(III) ions and Pb(II) ions is shown in Figure S2. The difference is based on the fact that both ions have different interactions, hydration, and ion chemistry. The Cr(III) ions have a higher hydration energy and are bound tightly in hydration shells, and they can easily exchange water molecules, whereas Pb(II) ions have a lower hydration energy and create bulky hydration complexes, such that they can be effectively hindered in the nanopores [60]. Hence, Pb(II) ions have a higher rejection than Cr(III) ions, i.e., Pb^+2^ > Cr^+3^.

## 6. Limitations and Comparison with Other Membranes

DESs are synthesized from readily available, cheap components, and the methodology consists of simple mixing operations. There are no complex purification steps, which leads to cost-effectiveness and energy efficiency. The recyclability, thermal stability, and low volatility of DESs paved the pathway toward their applications in large-scale processing. Also, the industrial applicability can be assessed by techno-economic evaluations and pilot-scale testing, which is a limitation of this work. The removal of chromium and lead ions has been widely reported via absorption, adsorption, and membrane separation, but there is a trade-off between the flux and the rejection of the metal ions. Table 2 provides an assessment of the DM-Zn-PDC@MOF membrane through a comparison of its flux and metal ion rejection with existing membrane systems under diverse conditions, which have been used to eliminate heavy metals. The membrane achieves exceptional performance through its flux capacity, which reaches 871.8 L m^−2^ h^−1^ and outmatches most existing systems, except for the Amino-Zr-MOF ceramic membrane, which achieves higher permeability through its capacity to reach 1100 L m^−2^ h^−1^. DM-Zn-PDC@MOF membrane shows a limited capacity to extract Cr ions (10.9%) and Pb ions (24.5%) when it is compared to 85% Cr ion removal via a PES/ER-blend membrane and 86.3% Pb ion removal via a PSf/PNU-1 membrane based on phytic acid-functionalized NH_2_UiO-66 nanoparticles and chitosan-PES/PP, which achieves a Cr ion extraction of 75%, but it has higher flux than chitosan-PES/PP and PES/ER-blend membranes and has higher permeability than PSf/PNU-1 membrane. The flux capacity provides an essential benefit for industrial applications, as the industry requires the processes to deal with high volumes, and flux is a significant factor. The DM-Zn-PDC@MOF membrane shows an excellent permeability with a reduced selectivity, which makes it suitable for water treatment facilities in which high concentrations are not required, and it can be used where the permeate can be further treated to increase purity, also the retentate can be recycled and subjected to resource recovery. Furthermore, the rejection can be increased using post-treatment techniques and adjusting the pore size of the membrane.

## 7. Conclusions

The DES-mediated MOF (DM-Zn-PDC@MOF) was successfully synthesized using a rational design approach, and it was integrated into a polysulfone (PSf) membrane matrix to fabricate novel mixed matrix membranes (MMMs). This was achieved through a straightforward blending approach, where different loadings of the MOF (5, 10, 15, and 20 wt% relative to PSf) were utilized, followed by phase inversion to produce a controlled thickness using a doctor blade. This simple fabrication process facilitated the effective dispersion of the filler and enabled the adjustment of membrane characteristics. The physicochemical properties of the membrane were greatly impacted by the addition of DM-Zn-PDC@MOF. The water contact angles stayed between 60 and 65 degrees compared to the pristine membrane, suggesting increased hydrophilicity. The leaching studies confirmed acceptable filler–polymer compatibility, with a maximum leaching ratio of only 4.31% observed for membrane M-2, whilst the pure water uptake increased significantly, reaching a maximum of ~200% for membrane M-3. These findings show that PSf and the MOF filler have a good level of interfacial adhesion. The pure water flux increased dramatically as the MOF loading increased, from 102.8 L m^−2^ h^−1^ for the pristine membrane (M-0) to 971.5 L m^−2^ h^−1^ for M-4, and the pure water permeability improved from 12.9 L m^−2^ h^−1^ bar^−1^ to 121.4 L m^−2^ h^−1^ bar^−1^. Despite a slight improvement in porosity, a significant increase in the pore size was noted, which directly increased the flux and overall heavy metal adsorption for further recovery. Despite this, there was a distinct pattern in the rejection pattern of high-value heavy metals: membranes containing more DM-Zn-PDC@MOF (M-4) showed better adsorption-assisted rejection (10.9% for Cr(III) ions and 24.5% for Pb(II) ions). Regarding the permeation performance during metal ion filtration, M-3 had the highest flux for Cr(III) solutions (526.8 L m^−2^ h^−1^), while M-4 had the highest flux for Pb(II) solutions (871.8 L m^−2^ h^−1^). In order to maximize both flux and heavy metal rejection, future research should concentrate on optimizing the balance between the pore structure, adsorption capacity, and hydraulic performance. Large-volume processing and metal concentration in the retentate are enabled by the current membrane’s high flux, which transforms diluted contaminated streams into concentrated feedstock for further recovery processes. This establishes the foundation for upcoming advancements targeted at effective metal recovery and the creation of workable recycling plans.

## Figures and Tables

**Table 1:** Compositions of the synthesized membranes (by wt%).

Membrane	DES-Mediated MOF (Filler)	Polysulfone	NMP Solvent
M-0	-	20.00	80.00
M-1	0.25	20.00	79.75
M-2	0.5	20.00	79.5
M-3	0.75	20.00	79.25
M-4	1.00	20.00	79.00

**Table 2:** Comparison of the current study with already reported relevant works.

Membrane Type/System	Flux (L m^−2^ h^−1^)	Permeability (L m^−2^ h^−1^ bar^−1^)	Cr Ion Removal Efficiency (%)	Pb Ion Removal Efficiency (%)	Reference
Amino-Zr-MOF Ceramic membrane	1100	-	-	61.4	[61]
Chitosan-PES/PP	9.3	-	75	-	[62]
PES/ER-blend membrane	12.17		85		[63]
PSf/PNU-1	-	66.5	-	86.3	[64]
Poly(butylene adipate-co-terephthalate) membrane	-	-	6	-	[65]
Commercial NF270 membrane	-	-	-	33.8	[66]
DM-Zn-PDC@MOF membrane	526.8 (Cr sol.) & 871.8 (Pb sol.)	65.9 (Cr sol.) & 109 (Pb sol.)	10.9	24.5	This work

## Data Availability

The data will be made available upon request.

## Supplementary Materials

The following supporting information can be downloaded at https://www.mdpi.com/article/10.3390/membranes16060205/s1, Figure S1: XRD spectra of membranes; Figure S2: Comparison of the rejection of Cr(III) and Pb(II).
