# Supplementary material for: Rational Design of Deep Eutectic Solvent-Mediated MOF-Based Membranes for the Recovery of Pb(II) and Cr(III) Ions Toward a Circular Economy
Source: Membranes (Basel). 2026 Jun 10;16(6):205. doi: 10.3390/membranes16060205 (PMC13302844; doi:10.3390/membranes16060205)
Supplement: Supplementary file 1 [file membranes-16-00205-s001.zip › membranes-4179249-supplementary.pdf]

Rational Design of Deep Eutectic Solvent-mediated MOF based Membranes for the Recovery of Pb(II) and Cr(III) ions Toward a Circular Economy

Saif-ur-Rehman, Urooj Ahmad, Muddasar Jamal, Arafat Husain, Bart Van der Bruggen and Ali H. Al-Marzouqi

X-ray diffraction of membranes

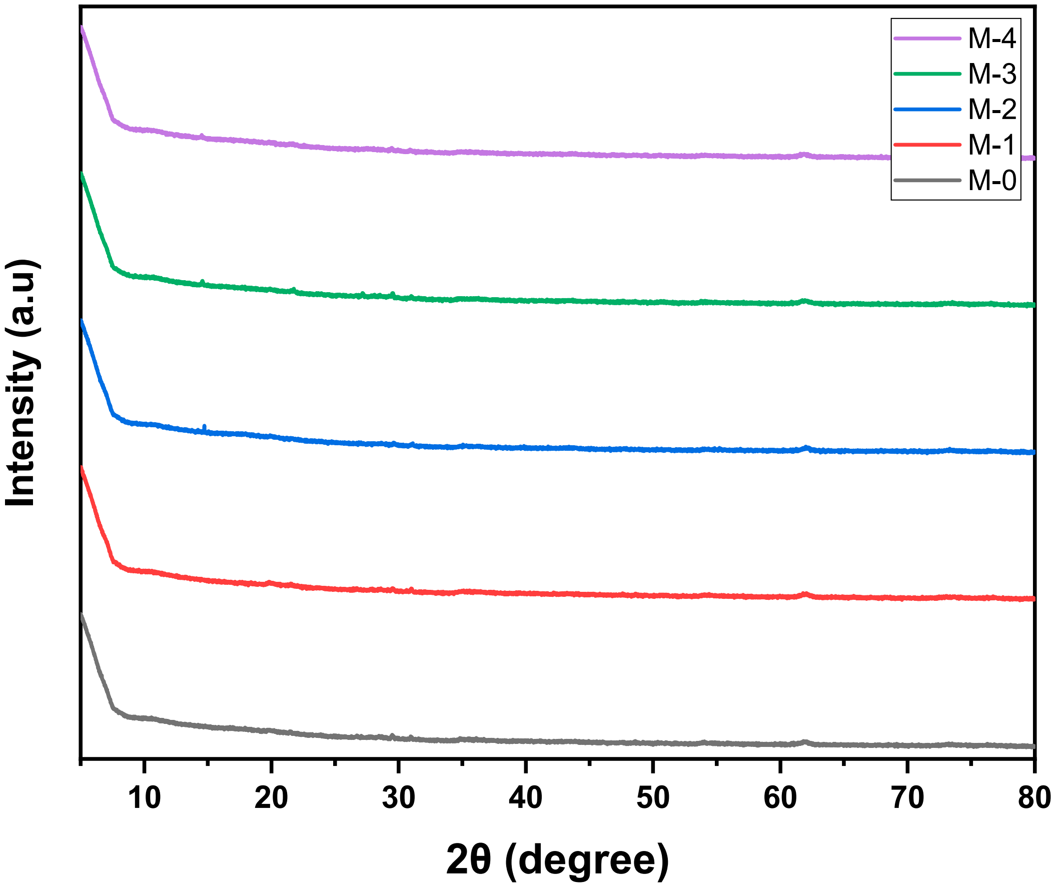

Figure S1: XRD spectra of membranes

Removal of Cr(III) and Pb(II)

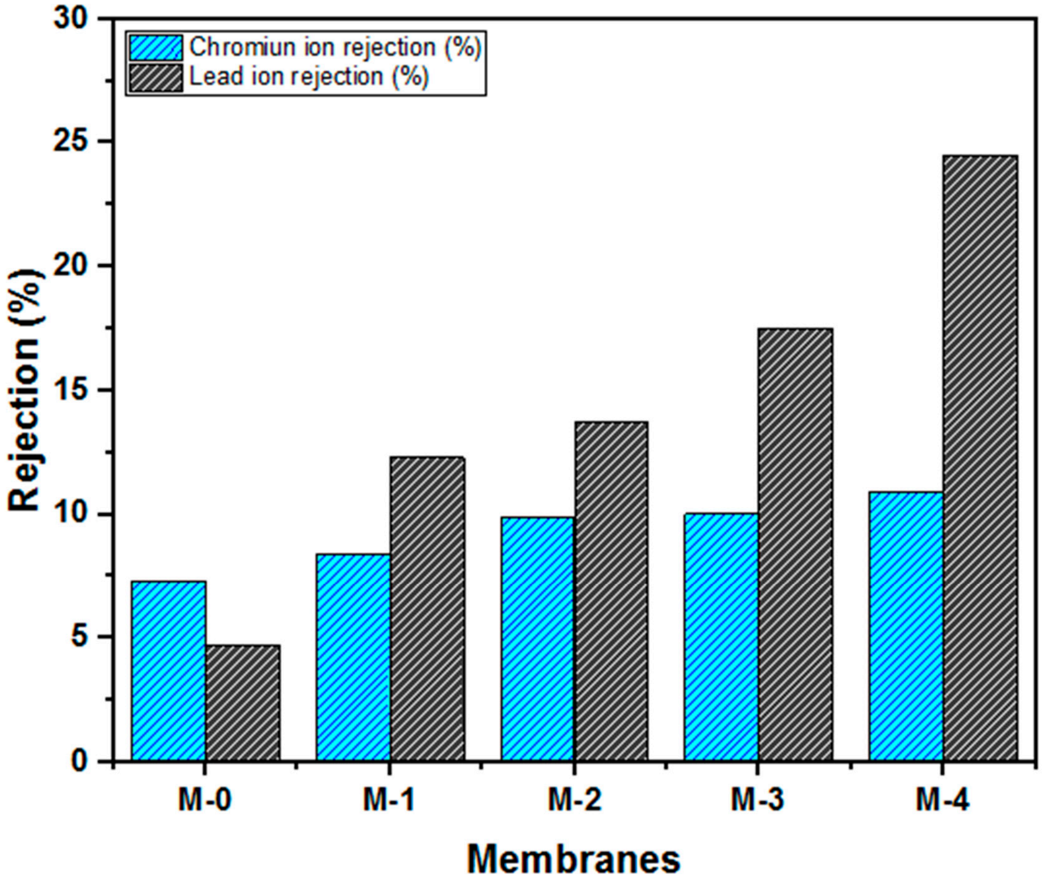

Figure S2: Comparison of the rejection of Cr(III) and Pb(II)
